# New Approach Methods to Evaluate Health Risks of Air Pollutants: Critical Design Considerations for In Vitro Exposure Testing

**DOI:** 10.3390/ijerph17062124

**Published:** 2020-03-23

**Authors:** Jose Zavala, Anastasia N. Freedman, John T. Szilagyi, Ilona Jaspers, John F. Wambaugh, Mark Higuchi, Julia E. Rager

**Affiliations:** 1 MedTec Biolab, Inc., Hillsborough, NC 27278, USA; jose@medtecbiolab.com; 2 Department of Environmental Sciences and Engineering, Gillings School of Global Public Health, The University of North Carolina at Chapel Hill, Chapel Hill, NC 27599, USA; stasfree@live.unc.edu (A.N.F.); szilagyi@email.unc.edu (J.T.S.); 3 Department of Pediatrics, School of Medicine, The University of North Carolina at Chapel Hill, Chapel Hill, NC 27599, USA; ilona_jaspers@med.unc.edu; 4 Center for Environmental Medicine, Asthma, and Lung Biology, The University of North Carolina at Chapel Hill, Chapel Hill, NC 27599, USA; 5 Curriculum in Toxicology, The University of North Carolina at Chapel Hill, Chapel Hill, NC 27599, USA; 6 The Institute for Environmental Health Solutions, Gillings School of Global Public Health, The University of North Carolina at Chapel Hill, Chapel Hill, NC 27599, USA; 7 Center for Computational Toxicology and Exposure, Office of Research and Development, United States Environmental Protection Agency, Durham, NC 27709, USA; wambaugh.john@epa.gov; 8 Center for Public Health and Environmental Assessment, Office of Research and Development, United States Environmental Protection Agency, Durham, NC 27709, USA; higuchi.mark@epa.gov

**Keywords:** air pollution, in vitro, inhalation, mixtures, new approach methods, toxicology, risk assessment

## Abstract

Air pollution consists of highly variable and complex mixtures recognized as major contributors to morbidity and mortality worldwide. The vast number of chemicals, coupled with limitations surrounding epidemiological and animal studies, has necessitated the development of new approach methods (NAMs) to evaluate air pollution toxicity. These alternative approaches include in vitro (cell-based) models, wherein toxicity of test atmospheres can be evaluated with increased efficiency compared to in vivo studies. In vitro exposure systems have recently been developed with the goal of evaluating air pollutant-induced toxicity; though the specific design parameters implemented in these NAMs-based studies remain in flux. This review aims to outline important design parameters to consider when using in vitro methods to evaluate air pollutant toxicity, with the goal of providing increased accuracy, reproducibility, and effectiveness when incorporating in vitro data into human health evaluations. This review is unique in that experimental considerations and lessons learned are provided, as gathered from first-hand experience developing and testing in vitro models coupled to exposure systems. Reviewed design aspects include cell models, cell exposure conditions, exposure chambers, and toxicity endpoints. Strategies are also discussed to incorporate in vitro findings into the context of in vivo toxicity and overall risk assessment.

## 1. Introduction

### 1.1. Introduction to Air Pollution Health Impacts

Air pollution negatively affects human health worldwide, with ambient air pollutants estimated to be responsible for approximately 3.7 million deaths annually [1,2]. This estimate equates to 6.7% of all mortalities worldwide, with air pollution-relevant deaths resulting from respiratory diseases (e.g., lung cancer, respiratory infections, etc.) and other related diseases (e.g., cardiovascular disease) [1]. Air pollutants can be split into two main categories based on emittance and/or formation sources: primary and secondary air pollutants. Primary air pollutants are those emitted directly into the atmosphere, including industrial and biogenic processes; while secondary air pollutants are those that are formed within the atmosphere. In terms of chemical composition, air pollution represents a complex mixture of both gases and particulate matter (PM). Both gases and PM are recognized to pose a threat to human health [3] and both can induce varying toxicity over time as they react under atmospheric influences [4,5,6,7].

It has been estimated that up to 87% of the world’s population lives in areas that exceed the World Health Organization’s (WHO) Air Quality Guidelines for PM_2.5_ (PM with diameter of less than 2.5 µm), estimated to contribute to 2.9 million deaths per year [8]. Gases within the atmosphere are also present at deleterious levels, with ozone, for example, estimated to contribute annually to 217,000 deaths [8]. Though the general public is impacted by air pollution exposure, susceptible populations such as the elderly, pregnant women, children, and those with certain preexisting diseases can be at even higher risk of related morbidity and mortality [9]. As an example, increased levels of outdoor ozone, sulfur dioxide, and carbon monoxide have been associated with a higher risk of cardiovascular-related hospitalizations among patients aged 65 and older [10]. Underlying asthma can also cause increased susceptibility to air pollutant-induced effects, with asthmatic individuals shown to experience exaggerated responses to ozone, among others [2]. Other social factors such as health disparities, including poor nutrition, low socioeconomic status, and race, are also associated with increased risk to air pollutant-induced adverse health effects [11].

Air pollution represents a dynamic and complex mixture of chemicals that can occur as individual constituents, as well as co-occur in a limitless number of combinations, making it difficult to accurately evaluate and quantify health risks attributable to air pollution components. Furthermore, it is impossible to test health impacts of all chemicals present in air pollution under atmospheric aging conditions. To address these critical limitations, new methods based on in vitro and in silico approaches are needed to more accurately evaluate air pollution toxicity and determine the contribution of the various components to the overall toxicity. When designed and interpreted using rigorous and scientifically sound approaches, data from these methods have the potential to improve health risk characterizations, resulting in regulatory action that more effectively protects public health.

### 1.2. Methods to Evaluate Air Pollution Health Impacts

There are many types of study designs that can be used to evaluate the health effects associated with air pollutant exposure, including epidemiological, controlled human, animal, and in vitro-based studies. A high-level overview of these study designs in air pollution research is discussed here. An introduction to enhancing such air pollutant evaluations through the incorporation of alternative methods, including in vitro (cell-based) models, is also discussed.

#### 1.2.1. Epidemiological Studies in Air Pollution Research

The goal of environmental epidemiology research is to better understand relationships between environmental factors and their potential impact on public health [12]. In general, these types of studies use statistical methods to relate measures of environmental exposures to disease outcomes based on observational data, rather than experimental data. Epidemiological studies on air pollution include (i) short-term designs, in which air pollution episodes are associated with acute health outcomes; as well as (ii) long-term designs, in which chronic air pollution measures are associated with incidences of both acute and chronic health outcomes [12]. These study designs have been used to characterize associations between various air pollution exposure conditions and multiple disease outcomes.

A historical example of epidemiological research was conducted surrounding the 1952 London Fog, one of the most referenced fog events in the twentieth century that led to the public’s understanding of the dangers of air pollution [13]. In that instance, a study published two years after the event found a relationship between the number of deaths per day and the rapid increases in sulfur dioxide and smoke concentrations [14]. In terms of long-term effects, this study also found that the daily number of deaths did not return to normal until about two weeks after the incident occurred [14]. Another historical example surrounded the 1948 Donora Smog event in Pennsylvania, encompassing five days of heavy smog resulting from poor implementation of smoke control measures [15]. Pollutant exposure conditions were associated with increased rates of mortality due to cardiovascular disease, particularly in subjects that had previous reports of heart or respiratory disease [15]. These historical examples, among others, laid the foundation for the current breadth of epidemiological research supporting the role of air pollution exposures in human health and disease. Epidemiological investigations in the field of air pollution, to date, have been conducted using various study designs, study cohorts, climate conditions, and air pollutant categories, and have been previously reviewed [16,17,18,19].

#### 1.2.2. Controlled Human Studies in Air Pollution Research

In some instances, human volunteers can be used to evaluate the health impacts of select air pollutants. This has been done, for instance, in the evaluation of diesel exhaust particles [20] and ozone using controlled human exposure chambers [21,22]. These study designs typically involve humans that are exposed to controlled single pollutants or pollutant mixtures either for short one-time durations or repeat conditions that would induce transient adverse effects that pose no long-term damage. Endpoints that are typically evaluated include general health symptoms, physical examination results, pulmonary function test results, and electrocardiographic indices. More molecular/cellular/chemical-based endpoints can also be evaluated using collected biological samples, which can include breath condensate, blood, urine, sputum, and bronchoscopy samples (e.g., bronchial brushing, bronchoalveolar lavage fluid, and/or endobronchial biopsy) [20]. Obtaining data from humans exposed in such controlled environments is advantageous since accurate characterization of exposure conditions and health responses in humans can be used for comparison and validation of data obtained from animal studies. Though it is important to consider that these types of studies are resource-intensive, require institutional review board approval, and have inherently complex ethical considerations.

#### 1.2.3. Animal Studies in Air Pollution Research

The toxicological effects caused by air pollutant exposures can be evaluated in more controlled environments through the use of animal testing. Rats and mice are the most common model, though other animals such as nonhuman primates, dogs, and rabbits have also been used to evaluate air pollution toxicity [23]. Air pollutant exposures can be conducted in animals using acute, subchronic, and chronic study designs. Acute designs can involve for example, a single day exposure, two-day exposures, or daily exposures that span 5–7 days/week across ≤14 days. Subchronic designs consist of similar daily exposures, but are maintained for up to 90–180 days; and chronic designs span between 180 days to two years [23,24]. Many types of toxicological outcomes can be observed from animal studies, including overall mortality and gross abnormalities, organ and tissue-level effects, and molecular-level effects.

There are several types of dosing routes in which animals can be exposed to gaseous or PM-based pollutants in a laboratory setting. These routes include gavage, aspiration, instillation, and inhalation [25]. Oral gavage represents a route in which chemical substances are administered to animals using a small plastic feeding tube passed through the nose or mouth and into the stomach [26]. Aspiration and instillation exposure routes are similar in that they involve the introduction of substances to the throat. Aspiration involves the introduction of substances into the region of the pharynx whereas instillation requires substances to be introduced directly into the trachea [27,28]. These methods have become widely used as they allow for greater control over dosage and location compared to other exposure routes, such as inhalation, though these routes have certain limitations. For example, aspiration/instillation exposure designs can omit certain chemical groups and may introduce differences in chemical deposition in comparison to inhalation exposure designs. These approaches remain common routes of exposure used to test air pollutant toxicity [29,30,31] due to many advantages, including increased feasibility of collecting and evaluating various test atmosphere samples, the ability to more fully characterize chemical composition of the sample, and increased feasibility of in vivo testing to provide reproducible results.

Exposing animals via inhalation represents another route commonly used in the in vivo testing of air pollutants, and can occur using whole body, head only, or nose only designs. Whole-body inhalation designs allow for potentially large numbers of animals to be exposed either simultaneously or separately, wherein animals are not restrained or anesthetized during exposure [24]. Limitations surrounding whole body inhalation designs include the potential for highly variable dosing across animals and competing exposure routes (e.g., dermal and ocular exposures) [24]. Head- or nose-only inhalation designs allow for repeat exposure conditions that are more consistent across animals, though may introduce stress as animals are restrained [24]. An advantage to using the inhalation exposure route is that these conditions reflect those that occur when inhaling air pollutants.

In general, there are advantages and limitations to using animals in the testing of air pollutant toxicity. A notable advantage is the ability to incorporate many types of controlled models. For example, genetically modified models can be used to evaluate disease mechanisms and potential interindividual disease susceptibility. Other exposure models can include models of disease (e.g., asthma) and models at critical periods of development (e.g., in utero) [32,33,34]. Additionally, animal models have the inherent advantage of providing the opportunity to evaluate potential systemic effects (e.g., cardiovascular toxicity) as well as other toxicity responses throughout multiple organs of the body [23,24]. Potential limitations in using animal models include physiological and genetic differences between animals and humans, which can impact air pollutant dosimetry and associated toxicity [35,36,37]. Animal study exposures can also require large amounts of time and resources [38] and require important ethical considerations [39]. Animal models have historically been used as the standard in toxicological assessments of air pollutants; though much attention has been placed on reducing reliance upon animal testing in recent years [40,41].

#### 1.2.4. New Approach Methods in Air Pollution Research

To address the increasing need to implement more efficient toxicity screening of air pollutants, new approach methods (NAMs) are being expanded upon and represent an area within inhalation exposure and toxicity research that is rapidly growing. The term “NAMs” represents a relatively new research classification that was introduced within the past five years, with definitions expanding to broadly include new experimental and computational approaches that can more rapidly inform chemical risk assessments [42,43,44]. NAMs; therefore, include in vitro models, which often require less time and resources to screen chemicals for toxicity. This is particularly important for air pollutant research, as many airborne chemicals and mixtures are currently lacking data required for hazard assessment. A few reviews have been published previously on the topic of in vitro models in inhalation toxicology [45,46], including some that were recently published [47,48]. Here, we contribute to this expanding body of literature by reviewing critical elements of study design to incorporate when planning and executing in vitro methods in air pollution research, with the ultimate goal of incorporating these NAMs into chemical hazard and risk assessment applications.

### 1.3. Purpose of the Current Review

The specific aim of this review is to outline important design parameters to consider when using in vitro methods to evaluate air pollutant toxicity, with the goal of ultimately providing increased accuracy, reproducibility, and effectiveness when incorporating in vitro data into human health and chemical risk assessments. Updated resources are currently limited for scientists designing experiments using in vitro methods to evaluate air pollution toxicity. This review uniquely addresses this resource gap by covering aspects of experimental design and “lessons learned” from the authors’ first-hand experiences, as well as issues raised within the published literature. Current data gaps and limitations surrounding experimental, biological, and computational aspects are also discussed. The current review therefore serves as an important resource for toxicologists, exposure scientists, and risk assessors that are interpreting findings from published inhalation toxicology studies, as well as planning future experimentation to more accurately and efficiently evaluate air pollutant-induced toxicity.

## 2. In Vitro Study Design Considerations

When designing an in vitro study to evaluate air pollutant toxicity, it is important to carefully consider experimental aspects that will play large roles in data accuracy and reproducibility. It is also advantageous to maximize the value of any in vitro-derived results by ensuring the translatability of findings into the context of in vivo toxicology and overall chemical safety evaluations. With these goals in mind, critical design aspects are reviewed here and include cell models, cell exposure conditions, exposure chambers, and toxicity endpoints. 

### 2.1. Cell Models

Many types of cell models can be used to evaluate air pollution toxicity. Models include various monoculture cell lines, cells from human donors, including those with underlying diseases, and other emerging models that are currently being integrated into air pollution studies. As reviewed here, each model has inherent advantages and disadvantages (Table 1); though when used with potential limitations in mind, can impart valuable information towards understanding the toxicity resulting from air pollutant exposures.

#### 2.1.1. Monoculture Cell Lines

The types of cells that are commonly used in air pollution research largely consist of cells derived from the respiratory system. Cell line selections for inhalation toxicology research have previously been summarized and reviewed [49]; and as such, this section provides a high-level overview of pertinent cell lines. Some of the most widely used immortalized cell lines represent those that are derived from human tumors or transformed from normal primary cells. A widely used cell line derived from a human tumor is the A549 cell line, derived from adenocarcinomic alveolar basal epithelial cells. A549 cells are still commonly used in air pollution toxicity studies, as they are easy to grow and maintain, provide toxicity results that are reproducible, and can remain viable in conditions that may cause cytotoxicity in other more sensitive cell lines (e.g., field site studies). Furthermore, A549 cells have important characteristics that parallel the in vivo airway epithelial lining, including their ability to mimic airways surface tension by secreting surfactant [50]. For these reasons, A549 cells have been used to evaluate toxicity resulting from exposure to a multitude of pollutants specifically including 1,3-butadiene [6], formaldehyde [51], ozone [52], and complex atmospheric mixtures [7,53]. A549 cells have also notably been used in high-throughput screening initiatives (e.g., ToxCast/Tox21) [54]. However, when using such a tumor-derived cell line to inform toxicity responses that occur in animals or humans without cancer, it is important to remember that differences exist between cancer and non-cancer cells. For example, it is well established that cancer cells can exhibit differences in genetic sequences, epigenetic profiles, and other underlying molecular patterns in comparison to non-cancer cells, which can impact the ways in which cells respond to toxicant exposures.

Additional examples of immortalized and/or transformed cells from the respiratory tract that are also commonly used in the laboratory setting include human cells lines (e.g., BEAS-2B, Calu-3, and 16HBE14o-) as well as rodent cell lines (e.g., LA-4 and MHS), among others. For example, the BEAS-2B cell line consists of immortalized human bronchial epithelial cells that have been infected with an SV40/adenovirus 12 hybrid and cloned. These cells can differentiate into squamous cells in response to certain substances, which make them amenable for screening chemicals that may induce or affect cell differentiation processes potentially relevant to carcinogenesis [55]. Studies using BEAS-2B cells have identified alterations in cell differentiation signaling in response to PM_2.5_ [56], indoor molds [57], and diesel exhaust particles [58].

When deciding which cell type to use in an in vitro study design, it is important to consider the advantages and limitations surrounding cancer vs. primary cell lines, as well as the applicability of the respiratory region in which the cells were derived. As mentioned above, cancer cells lines are more robust and can therefore be more amenable to varying exposure conditions; and transformed cell lines can be more indicative of responses from healthy individuals [49]. Furthermore, the region of the respiratory tract in which the cells were derived is important to consider. It may be more applicable to use cells from the upper respiratory tract (e.g., nasal, pharynx, and larynx cells) when evaluating air pollutants that are more volatile and are mostly absorbed within the upper respiratory tract after inhalation [59,60]. Conversely, cells from the lower respiratory tract (e.g., tracheal, bronchial, and alveolar cells) may be more suitable for evaluating air pollutants that are less volatile and reach the lower respiratory region [61]. Other cells involved in respiratory tract signaling, including immune cell populations, such as macrophages, can also be used to evaluate potential responses in immune signaling relevant to air pollution [49]. For example, the RAW264.7 is a commonly used macrophage cell line that has been employed to demonstrate the inflammatory effects of air pollution on macrophages in vitro [62,63,64]. When properly designed, monoculture techniques can contribute valuable information surrounding potential toxicity and disease mechanisms associated with air pollutant exposure.

#### 2.1.2. Cells from Human Donors

Studies have leveraged the use of cells collected directly from human donors, including nasal and bronchial epithelial cells, and lung macrophages [49,65,66]. Advantages of using cells collected from humans include the ability to perform analyses on a specific subpopulation of interest. For example, our research group has evaluated primary differentiated human nasal epithelial cells obtained from volunteers, selecting according to different age groups (i.e., between the ages of 20–27 and 55+) [67]. These data provide insight for important age-associated differences between epithelial responses to air pollutants [67]. Obtaining cells from human donors also allows for the identification of toxicity responses and trends in disease susceptibility that may be dependent upon sex (i.e., sexually dimorphic). For instance, increased expression of interleukin-6 (IL-6) and interleukin-8 (IL-8) have been found in nasal epithelial cells derived from males exposed to ozone, while this trend was not apparent in females [68]. A potential limitation surrounding the use of cells from human donors includes the identification of study participants that meet specific study inclusion criteria at high enough enrollment rates, ensuring study feasibility [67]. Cells from donors can also exhibit limited life spans in comparison to cell lines [49].

To address potential limitations of human donor cultures, commercially available airway tissue models have been developed. Examples of commercial airway tissue models that are currently used in research include the MucilAir (Epithelix Corp., Geneva, Switzerland) and the EpiAirway (MatTek Corp, Ashland, Massachusetts, USA) systems. Both the Mucilair and EpiAirway systems are 3D tissue models reconstituted using primary human respiratory epithelial cells, spanning nasal, bronchial, and/or tracheal cells [69,70]. They have been engineered to represent models of the human respiratory tract with potentially increased biological relevance as they are prepared by collecting cells from single or pooled donors that can vary in health status, ranging from healthy individuals to smokers to individuals with disease pathologies (e.g., asthmatics, chronic obstructive pulmonary disease, etc.). These cultures have been designed with improved physiological relevance as they consist of multiple layers of cells organized using engineered support systems, such as physical scaffolds or bioreactors that control nutrient and waste product exchange [71]. The cells are then cultured at an air–liquid interface (ALI) and shipped “ready-to-use” to research laboratories. Previous studies have tested these systems to evaluate mechanism of toxicity associated with air pollutant exposures [53,72,73].

More generally speaking, the accessibility and availability of the commercial airway tissue models are important for in vitro studies because they can incorporate multiple cell types, and allow for the identification of toxicity responses across the respiratory system [74], which is not possible using monocultures. However, when including multiple cell types in a study, it is important to consider the varying baseline expression and toxicity response profiles that can be present across multiple cell types [53,75,76]. These variable profiles can make it difficult to determine which cell type is inducing specific biomarkers or other toxicity responses associated with air pollutants. Although human donor cultures, such as MucilAir and EpiAirway models, are promising and allow for a broader analysis of air pollutant exposure effects, more peer-reviewed studies comparing responses between these models are necessary to expand our understanding of how these models can further inform the existing body of evidence on air pollution toxicity. Our research group has recently assessed the biological response variability in EpiAirway cells vs. A549 cells after ALI exposure to various air pollutants [53]. Results showed that the EpiAirway model was more toxicologically resistant compared to A549 cells when investigating changes in cell viability and cytokine secretion after exposure at the ALI [53]. These initial comparative analyses demonstrate the need to further evaluate potential differences in model sensitivity that are important to understand when designing and implementing in vitro screening of air pollutants.

#### 2.1.3. Lung-On-A-Chip Models

Lung-on-a-chip represents an emerging model that can be used in the evaluation of air pollution toxicity, which involves the culturing of respiratory epithelial cells on a flexible polymer scaffold designed to more accurately recapitulate conditions in vivo [77]. This technology offers several advantages over traditional in vitro models of lung. Common to other organ-on-a-chip technologies are the use of microfluidics to simulate blood flow [77]. This allows the continuous replenishment of nutrients and simultaneous removal of waste, unlike the standard, static culture media conditions which accumulate nutrients and waste until the media is refreshed manually [78]. Lung-on-a-chip often incorporates two compartments: one lined with endothelial cells purged with media and one lined with respiratory epithelial cells and exposed to air, generating an ALI [79]. This model; therefore, allows researchers to study more histopathologically-driven endpoints, such as pulmonary edema, by quantifying vascular leakage [77], and asthma, by measuring goblet cell hyperplasia, cytokine hypersecretion, and ciliary function [80].

Lung-on-a-chip scaffolding also provides researchers with the ability to exert mechanical forces, such as stretching, to mimic the natural motions of a breathing lung [81]. The inclusion of these mechanical properties contributes to increased accuracy in modeling in vivo conditions for the purposes of hazard identification and risk evaluation. For instance, the addition of mechanical stress to simulate breathing sensitizes alveolar epithelial cells to the toxic effects induced by silica nanoparticles and *Escherichia coli* [81]. Breathing lung-on-a-chip models have also been used in conjunction with other organs to form a body-on-a-chip [82]. As proposed, body-on-a-chip would link multiple organ-on-a-chip models with a common microfluidics system to accurately recapitulate a complete in vivo system [83,84]. A shared “circulatory system” would allow for the evaluation of multiple influences, including route of exposure, pharmacokinetics, endocrine signaling, and organ interaction, for toxicological assessments using one model [83,84]. In the study of air pollutants, for instance, the entire system could be exposed by “breathing” the compound of interest, which passes through the ALI across alveolar epithelial cells and interacts with the other organ systems through the shared microfluidics. However, this task would be challenging when trying to couple the lung-on-a-chip to a pollutant generation system, suggesting that more extensive research and development is currently needed. The lung-on-a-chip model has also been combined with micro-optical coherence tomography to quantitate cilia motion and mucociliary transport [85]. For chemical evaluation purposes, both lung-on-a-chip and body-on-a-chip models allow for increased physiological relevance in comparison to traditional in vitro techniques, while providing a more humane practice than those based on in vivo designs.

Lung-on-a-chip models are still being developed and improved upon and have certain limitations [86,87]. For instance, employed lung-on-a-chip models are highly dependent upon the types of cells that are seeded into the model itself; therefore, the aforementioned limitations inherent in the cells translate to the chip model. Additionally, there are continued discussions surrounding the accurate composition of artificial blood used in microfluidics. Further, these systems represent advances in methods to parallel in vivo tissue organization; though full recapitulation of tissue organization remains to be achieved through these approaches. Other limitations exist, particularly surrounding ease-of-use; though, lung-on-a-chip designs may be incorporated with increased feasibility in the upcoming years.

### 2.2. Cell Exposure Conditions

#### 2.2.1. Exposures Using Submerged Conditions

Cells are often exposed within the in vitro setting through the use of submerged conditions, wherein cells are exposed to chemicals of interest that have been dissolved in a liquid. Conventional in vitro exposure studies of airborne pollutants, for example, involve the addition of PM or PM extracts to the cell culture medium, or the bubbling of gases into the culture medium. Though with these study designs, the physical and chemical characteristics of tested substances may be altered (e.g., particles agglomerating or dissolving) when placed in solution, thus changing the exposure conditions that would occur in the air. Dosing of cells attached under submerged conditions is beneficial when individual chemicals are soluble in water or dimethyl sulfoxide (DMSO). This method becomes problematic when the testing of insoluble chemicals is needed, as well as airborne particulates and complex mixtures that vary in composition once dissolved in a liquid. Furthermore, the apical media inherently in submerged cell cultures can alter the respiratory epithelial cell phenotype, expression of critical genes, and barrier conditions for gases to contact the cells under evaluation [48]. Although cell-based assays using submerged conditions can still impart useful information surrounding air pollution toxicity, these limitations must be taken into account when designing and interpreting such studies. More realistic cell exposure conditions that evaluate toxicity resulting from gases, vapors, and aerosols incorporate ALI designs.

#### 2.2.2. Exposures Using Air–Liquid Interface Conditions

If the main route of exposure in humans is via inhalation for the particular chemical or particle of interest, then it is clearly advantageous to evaluate exposure effects in a similar fashion. It has become widely accepted that exposing cells at the ALI is advantageous to simulate an inhalation exposure [47,48]. Cells in the ALI condition are cultured on porous inserts that contain cell culture medium in the basolateral side to maintain viable cells, while the apical side is directly exposed to the air, permitting a direct exposure [88,89]. Cells exposed via ALI have demonstrated different toxicity responses in comparison to those exposed via submerged conditions [90,91]. As an example, diesel exhaust exposures have been found to induced greater inflammatory responses when conducted at the ALI vs. submerged conditions, in which samples were collected on a filter and resuspended in culture medium [92]. Although ALI exposures are used to better represent direct pollutant-to-cell interactions and can be reasonably regarded as an effective in vitro surrogate for inhalation, these studies are inherently more complex to carry out in comparison to traditional submerged conditions. Exposing cells at the ALI requires the use of specialized in vitro exposure equipment, as detailed in the next section.

### 2.3. ALI Exposure Chambers

**Figure 1:**
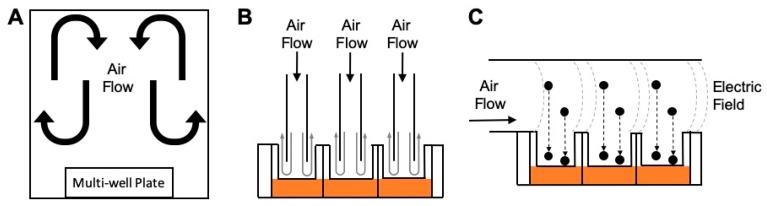
Schematic of in vitro exposure chamber systems. (**A**) An undirected flow system is shown with air circulating within the “box” housing a multi-well plate containing cells. (**B**) A perpendicular flow system is illustrated with nozzles that direct the air flow directly over the cells. (**C**) A horizontal flow system shows air flow moving across the cell culture inserts that are sitting below the flow path.

Various ALI in vitro exposure systems have been developed by multiple research groups, and few systems are currently available commercially. Some systems have been optimized for delivering either gases or particles onto cells effectively. For particle delivery, various mechanisms such as diffusion, sedimentation, thermophoresis, and electrostatic precipitation have been incorporated into these systems. Recent reviews have provided a summary of the existing ALI chamber systems and the state of the science [88,93,94]. In brief, these exposure chamber systems can be grouped into three categories, depending on the methods used to introduce air flow: (i) undirected flow, (ii) perpendicular flow, and (iii) horizontal flow. Figure 1 illustrates a basic schematic for each of these different types of in vitro chamber systems. Examples of these types of systems are described here, along with a discussion on exposure parameters and test controls.

#### 2.3.1. Undirected Flow

An undirected flow exposure system consists of a “box” where inserts are exposed to air that is set-up to circulate both above and around the cells. Because the air flow is undirected, these exposure chambers are typically used to expose cells to gaseous pollutants. This type of exposure system has been used by government agencies, academic institutions, and research institutions. For example, the U.S. Environmental Protection Agency (U.S. EPA) has used these types of systems since the early 1990s, consisting of incubator chambers that have been retrofitted to contain an isolated 28.3 L stainless steel enclosure containing two perforated shelves to support up to eight tissue culture plates of any format (i.e., six-, 12-, and 24-well). Air enters the chamber through a port on top at a flow rate of 20 L/min, and the air is randomly dispersed as it flows through the perforated shelves before exiting at the bottom [95]. An example chamber system using undirected flow within the academic setting was engineered at the University of North Carolina (UNC) at Chapel Hill and termed the Gas In Vitro Exposure System (GIVES). Similar to U.S. EPA’s Incubator Chambers, the GIVES consists of an 8 L chamber with a perforated platform. The inlet and outlet ports are located at the bottom and a flow rate of 1 L/min is used [95]. Both the U.S. EPA’s Incubator Chambers and the UNC GIVES are dynamic systems where the sampled air is flowing continuously.

Another example of an undirected flow system was generated at UNC-Chapel Hill for the purpose of evaluating electronic cigarette (e-cig) exposures [96,97]. This system comprises a 17.8 × 17.6 × 10.0 cm^3^ Plexiglas chamber with an inlet port for e-cig devices operated at conditions to mimic vaping. A brushless fan within the chamber circulates the incoming puff, allowing high doses (0.25–1.0 mg/cm^2^) to be delivered. Conversely, the Air–Liquid Interface Cell Exposure System (ALICE) System, produced by the German Research Center for Environmental Health, is an example of a static system with undirected flow that is used for aerosol delivery [98]. Here, a small box contains inserts at the bottom in a heated platform, while an inlet port is located on top. The inlet port uses an aerosol cloud generator (a pulse of air over a powder or liquid solution) to introduce an aerosol cloud that can deposit over the cells due to gravitational settling of the cloud [98].

#### 2.3.2. Perpendicular Flow

In a perpendicular flow system, a nozzle guiding the air flow directly over the cells is placed above each individual cell culture insert. Typically, the nozzle is positioned 1–5 mm above the cells. By doing so, the incoming air flow travels in a downwards direction, perpendicular to the cell surface. Two commercially available systems that use perpendicular flow are the Vitrocell Systems and the CulTex Systems. In these systems, the flow rate is typically operated at 2–10 mL/min/well, depending on the insert size [99]. These are dynamic systems that typically rely on diffusion and sedimentation forces to regulate particle deposition onto the cells, while a few other models use electrostatics to enhance this deposition. The Nano Aerosol Chamber for In Vitro Toxicity (NACIVT) is a similar system that was developed mainly for nanoparticle exposures, where electrostatics are used to enhance particle deposition [100]. In these systems, each individual cell culture insert is manually transferred onto a custom well inside the exposure system. In an effort to miniaturize the U.S. EPA’s Incubator Chambers, researchers have developed the Cell Culture Exposure System (CCES), which incorporates a perpendicular flow design coupled with thermophoresis to enhance particle deposition [95]. This system accommodates six- and 24-well plate formats which can be directly placed inside the system. This eliminates the individual handling of the inserts and facilitates the addition and removal from the exposure chamber. Similar to the CCES, the Fraunhofer Institute developed the ExpoCube system that also incorporates thermophoresis-enhanced particle deposition, where a 12-well tissue culture plate can be placed inside the system [101]. Other perpendicular flow systems focused on portability for on-site field testing have also been developed [102,103].

#### 2.3.3. Horizontal Flow

The horizontal flow system has been mainly used by the same family of chamber exposure systems that use electrostatics to enhance deposition. In these systems, the air flow occurs horizontally to the cells, with the culture insert positioned below the flow path. The cell culture inserts are placed in the deposition region where they can be subjected to an electric field as particles flow over the cells. An example system that includes this type of design is the Electrostatic Aerosol in Vitro Exposure System (EAVES), originally developed at UNC-Chapel Hill and used to evaluate diesel exhaust exposures [90]. The next iteration of this system was the modified-EAVES, which was used to study coarse PM and combustion particles [91]. This device has been further expanded upon into the development of the Gillings Sampler [104] and is now commercially available as the CelTox Sampler (MedTec Biolab, Inc) [105]. These horizontal flow systems, among others, represent important methods in which researchers can evaluate toxicity resulting from various air pollutant exposure conditions.

#### 2.3.4. Particle Deposition Forces

Understanding particle aerodynamics is essential when determining which chamber exposure system is the most appropriate for a particular study. Diffusion and sedimentation forces are natural forces exerted on the particle that vary depending on the particle’s diameter. Systems that rely on diffusion forces for particle deposition are appropriate when testing nano-sized (<100 nm) particles. For these nano-sized particles, as the diameter of a particle decreases, its associated diffusion velocity increases, resulting in increased deposition efficiency via diffusion [106]. Conversely, systems that rely on sedimentation forces (gravitational settling) are adequate when testing micron-sized (>1 µm) particles. For these micron-sized particles, the larger the particle diameter, the greater the gravitational settling velocity, thus the greater the deposition efficiency via sedimentation [106]. While these natural forces can be sufficient regulators of particle deposition in certain studies, poor performance is achieved for particles between 100 nm and 1 µm when systems simply rely upon natural deposition forces. In these instances, external forces can be used to enhance particle deposition. The use of thermophoresis (thermal forces) can improve deposition of nano-sized particles whereby the thermal forces are more dominant than diffusion and can help with particles slightly larger than 100 nm [107]. The use of electrostatic forces are applicable to all particle sizes, including PM_2.5_, an important range of particle sizes with reported association to many adverse health effects and thus the focus of major air regulatory guidelines [108,109].

#### 2.3.5. Exposure Parameters and Test Controls

The use of ALI in vitro exposures for screening the toxicity of air pollutants is now widely accepted as an effective in vitro surrogate for inhalation studies. As with any inhalation study in vivo, a matched in vitro clean air exposure should be conducted alongside the pollutant exposure to have a proper vehicle control for comparison. Additionally, a negative control is recommended whereby a multi-well plate containing matched cells grown on membrane inserts remain unexposed in the incubator. However, there is inconsistency in published research that prevents cross-study comparisons, meta-analyses, and finding consensus due to the lack of exposure guidelines and evident variability in how these systems are operated [99]. Even in the most basic parameter, such as the vehicle control, there exists variability among studies. Clean, filtered air is the standard vehicle control for ALI studies, yet studies have encountered issues in maintaining cell viability in samples exposed to clean air. To further limit variability and increase confidence in the reported findings, it is recommended to optimize control conditions in order to limit non-specific cytotoxicity resulting from clean air conditions. To achieve such conditions, our group has shown that regulating temperature (37 °C) and relative humidity (>75%) of the delivered air to the cells eliminates the cytotoxicity resulting from clean air exposures [99].

Within reported ALI exposure study designs, the methods and engineered exposure conditions should be clearly stated to allow for accurate interpretation of resulting data. Important engineering parameters to report include those that contribute towards determining adequate exposures and effectiveness of dose delivery. These parameters include: (1) air flow per well, (2) total flow rate, (3) temperature of the sampled air, (4) relative humidity of the sampled air, (5) the concentration in the air entering the ALI chamber, and (6) the dose delivered to the ALI cultures. In addition, it is important to describe the parameters of the pollutant generation system, the concentrations at the generation source, and the pollutant physicochemical properties [99]. These experimental parameters represent standard variables typically measured and reported in inhalation studies in vivo. By replicating these standards in vitro, it becomes more feasible to translate in vitro findings into the context of in vivo toxicology.

### 2.4. Toxicity Endpoints

**Figure 2:**
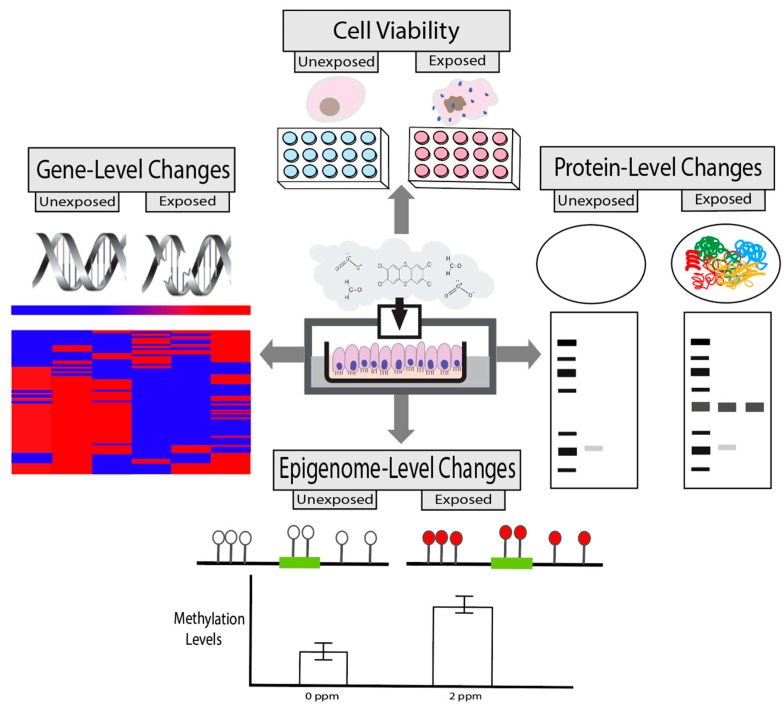
Types of toxicity endpoints relevant to air pollution exposure that can be evaluated through the use of in vitro models. A generic in vitro exposure condition held at an air–liquid interface, as an example, is shown in the middle, with various categories of toxicity responses that can be measured. For more information, see details within this review described under the overall categories of cell viability, gene-level changes, protein-level changes, and epigenome-level changes.

The types of toxicity endpoints that can be incorporated into in vitro screening efforts surrounding air pollution testing are ever increasing, in parallel with the growing number of technologies to evaluate biological mechanisms linking exposures to disease outcomes. Some of the most commonly incorporated toxicity endpoints are discussed here, and include measures of cell viability, gene-level changes, protein-level changes, and epigenetic-level changes (Figure 2). These endpoints are obtained using lab-based measures that are becoming increasingly reliant upon computational approaches for effective interpretations, as technologies and measured read-outs are providing increased depth of information at higher throughput [110,111,112,113].

#### 2.4.1. Cell Viability

Cell viability (or cell death) is a critical measure of toxicity that is needed in all in vitro study designs for quality control. The methods currently available to evaluate cell viability leverage measurable properties, such as the ability to quantitate select proteins/enzymes that are directly proportional to the number of viable cells in a given sample. These protein/enzyme measures are evaluated within exposed and unexposed cells to identify potential changes in cell viability from baseline conditions, as well as the conditions being tested. Positive controls should be included within the experimental design to accurately calculate the total percentage of cells that remain viable or have died. In general, methods of detecting cell viability are important quality control measures that are necessary for in vitro studies and are amenable for inclusion in high-throughput screening assays to ensure study quality and interpretability across a large scale [114].

A commonly used viability assay within the field of in vitro air pollution research includes the lactate dehydrogenase (LDH) assay. This colorimetric assay measures the amount of LDH released into cell culture supernatants when plasma membranes are damaged and this measure is directly proportional to the number of dead or damaged cells [115]. Cell viability can also be estimated using assays that rely upon cellular metabolic activity and ATP formation, including the CellTiter-Glo 3D, Water-Soluble Tetrazolium Salts-1 (WST-1), 3-(4,5-Dimethylthiazol-2-yl)-2,5-Diphenyltetrazolium Bromide (MTT), and Adenylate Kinase assays [116,117,118,119]. Alternatively, the AlamarBlue assay uses fluorescence to measure the reducing capabilities of living cells to convert resazurin to the fluorescent molecule, resorufin [120]. Similarly, in the Calcein AM cell viability assay, the Calcein AM permeates live cells which then is converted into a green-fluorescent calcein due to hydrolysis by intracellular esterases; and resulting stained cells can be quantified and correlated to cell viability [121]. There are many examples of studies using these types of assays to screen for cytotoxicity in air pollutant toxicity studies [4,6,7,51,53,122,123,124,125]. Consideration should be given to the use of these assays for investigations involving PM, as particles from diesel exhaust and soot have been shown to interfere with MTT and LDH signals at varying concentrations [126].

The Neutral Red Uptake assay is another type of test that detects the number of viable cells in a culture through the uptake of neutral red dye. Viable cells incorporate the dye into their lysosomes while non-viable cells do not carry out this dye-based incorporation [127]. The neutral red uptake assay has served as an industry standard since it has been validated and accepted by regulatory agencies, such as the Organisation of Economic Cooperation and Development (OECD) [128]. However, it is important to consider potential limitations in this assay when interpreting results from these study designs. For example, we have found that the neutral red dye can be absorbed by the porous membrane inserts that are commonly used in ALI designs, and thus be detected in the presence of viable cells. This can result in the false interpretation that cells are non-viable, when in actuality, cells are alive. It is therefore important to take into consideration these potential assay-specific limitations when interpreting findings from cytotoxicity assessments.

#### 2.4.2. Gene-Level Changes

There are several toxicity-associated responses at the gene-level that can be evaluated within in vitro air pollution studies, including those relevant to DNA damage (also known as genotoxicity) and gene expression. DNA damage can lead to mutations and genome instability, which represent common molecular events to evaluate when assessing the effects of chemical exposures using in vitro models [113]. DNA damage can be evaluated using many assay-based methods such as the comet, gamma H2A histone family member X (γ-H2AX), micronucleus (MN), and terminal deoxynucleotidyl transferase (TdT) dUTP nick-ends labeling (TUNAL) assays [113]. As an example, Rossner et al. analyzed DNA damage induced by gasoline engine emissions within BEAS-2B and the 3D MucilAir cell systems treated at an ALI [73]. Authors found that double strand breaks were induced within the BEAS-2B cells through use of the γ-H2AX assay [73]. A similar study found that engine emission exposures increased MN frequency in BEAS-2B cells [129]. Such indicators of genotoxicity are important to evaluate, as genotoxicity represents a potential key molecular event involved in chemical-induced carcinogenesis and other disease phenotypes [130,131].

Gene expression-level changes also represent a commonly investigated endpoint that is amenable to in vitro screening. Gene expression changes can directly result in protein expression changes, which are important to evaluate given that proteins are the ultimate regulators of cell function and overall health. Realtime quantitative reverse transcription PCR (qRT-PCR) is a common gene-specific method for investigating transcriptional changes. High-throughput technologies such as cDNA microarrays and RNA sequencing are also common platforms that are becoming increasingly utilized for understanding impacts across the genome [110,113]. For instance, we compared the toxicity of two different complex air pollutant mixtures representing those present within urban atmospheres using transcriptomic approaches coupled with in vitro air chamber exposure systems [7]. Through these and other studies, we have found that it is important to properly collect, lyse, and stabilize RNA from cell samples immediately post-experimentation using reagents specifically suited for RNA stabilization (e.g., TRIzol, RNAProtect, and similar reagents). These types of stabilizing reagents ensure that RNA remain intact and at high enough concentration to allow for genome-wide transcriptional assessments. Together, gene-level measures of toxicity are clearly important to evaluate in the context of air pollution responses and can be used to obtain important information surrounding disease mechanisms.

#### 2.4.3. Protein-Level Changes

Proteins are complex molecules that play critical roles to support various biological processes regulating cellular function, including those relevant to air pollution defense mechanisms and related disease processes. Protein measurement techniques can be employed within in vitro study designs by measuring activities/levels within and/or secreted outside the cells under evaluation. Within the context of air pollution studies, proteins involved in inflammation and immune response are commonly investigated. Example protein families that are highly relevant to these processes include the following: cytokines and growth factors [e.g., C-X-C motif chemokine ligands (CXCLs), C-C motif chemokine ligands (CCLs), interleukins (ILs), and tumor necrosis factor ligand superfamily members (TNFSFs)], and transcription factors [e.g., aryl hydrocarbon receptor (AHR), hypoxia inducible factor 1 subunit alpha (HIF1A), interferon regulatory factor 1 and 7 (IRF1 and IRF7), nuclear factor kappa B subunit 1 (NFκB1), and RELA proto-oncogene, NFκB subunit (RELA)], among others [132]. When secreted by epithelial cells in the respiratory tract, inflammatory proteins act in cell communication to recruit immune response cells (e.g., neutrophils) and other cells that respond to injury, such as macrophages [133]. Other proteins involved in air pollution responses can also be measured, including those involved in angiogenesis/vascularization, cancer, hormone regulation, oxidative stress, metabolism, and tissue injury signaling.

There are several methods that can be implemented to measure the concentration or activity of proteins within the context of in vitro studies. For example, studies have commonly employed Western blot and enzyme-linked immunosorbent assay (ELISA) technologies to evaluate the concentrations of proteins secreted intracellularly, as well as secreted during/after exposures into extracellular compartments [7,53,134,135]. Additionally, multiplex protein detection platforms such as Luminex allow the simultaneous quantification of multiple intra- or extracellular proteins to establish a protein expression profile after toxicant exposure in vitro. This technology is useful to measure lung inflammation as it can simultaneously quantify a panel of secreted cytokines [136]. In addition to expression, the testing of protein function may also be relevant to inhalation exposure, for instance as indicators of signal transduction or oxidative stress. As examples, enzymatic assays can be utilized in vitro to measure kinases (MAPK, JNK), proteases (MMPs), oxidases (NOXs), and metabolic activities (mitochondrial, CYP450s), among others, that may be altered after certain exposure conditions [137,138,139,140].

Careful considerations should be given to the use of ELISAs when exposing cells to combustion and engineered particles. Previous studies have shown that carbonaceous particles and engineered nanomaterials potentially interfere with the ELISA assay as the particles can absorb the proteins, thus preventing or decreasing detection and quantification [141,142,143]. Proteomics is also a growing research strategy which allows for the large-scale study of proteins, potentially resulting in the increased understanding of functional protein networks that can be modified by environmental exposures [110,113]. Proteomic studies within in vitro air pollution research remain limited; though future research could apply these strategies to more comprehensively examine the consequences of air pollution exposure conditions.

#### 2.4.4. Epigenetic-Level Changes

Epigenetic modifications represent toxicological endpoints that are more recently becoming integrated into air pollution health research. Epigenetic mechanisms influence the way in which genes are expressed without changing the underlying DNA sequence [112]. There are three main types of epigenetic modifications, namely, microRNAs (miRNAs), CpG methylation, and histone modifications. miRNAs are small, non-coding RNAs approximately 17-24 nucleotides in length that post-transcriptionally regulate the levels at which genes are expressed, either by directly binding to target mRNA molecules, recruiting chromatin modifying enzymes to target genes, and/or recruiting proteins to form ribonucleoprotein complexes [112]. CpG methylation represents the presence of a methyl group on the cytosine of a CpG dinucleotide within DNA [144]. CpG methylation can result in gene silencing through the attachment of methylation-sensitive DNA binding proteins and/or through interactions with certain histone protein modifications, decreasing access of transcriptional machinery to gene promoter regions [144]. Histone modifications represent the addition or removal of acetyl, methyl, or phosphoryl groups; ubiquitin; or small ubiquitin-like modifier proteins on histone [112]. These types of modifications have been associated with both increased and decreased expression of critical genes involved in disease progression [112]. Epigenetic modifications have the potential to persist through cell replication and become heritable; as such, these changes are vital towards understanding the potential long-term and multi-generational effects induced by toxicant exposure [145].

Previous in vitro studies have employed methods that assess epigenetic-level changes associated with air pollutant toxicity. As an example, our research group has used microarray techniques to measure genome-wide miRNA expression profiles in relation to gaseous formaldehyde exposure in human lung cells, identifying those involved in cancer and inflammatory processes [51]. We have also employed gene-specific approaches using methylation qPCR assays to identify increased promoter methylation of interferon regulatory factor 7 (*IRF7*) within nasal epithelial cells from smokers, which was then linked to enhanced susceptibility to viral infection using in vitro models [146]. To further understand the genome-wide landscape of CpG modifications associated with cigarette smoke, we also employed Illumina Methylation BeadChip technologies to identify 390 genes with significantly differential methylation between nasal epithelial cells from smokers vs. non-smokers, including an expanded number of genes involved in antiviral responses [147]. Similar to the aforementioned transcriptomic study designs, these studies highlighted the importance of adequate DNA/RNA stabilization and isolation protocols. In study designs that result in isolated DNA/RNA samples with high quality and purity, genome-wide epigenetic platforms can capture exposure-induced changes with higher sensitivity and accuracy. Together, these approaches can clearly contribute to the increased understanding of novel mechanisms underlying air pollutant-induced disease.

## 3. Placing In Vitro Findings in the Context of Animal and Human Exposure Conditions

Chemical risk evaluations have historically been reliant upon the use of data derived from in vivo studies. In the field of air pollution research, these have encompassed data from both animal studies as well as data from controlled exposure designs conducted in humans (e.g., ozone), in addition to epidemiological evidence [148]. However, as detailed in Section 1, there are important limitations surrounding these approaches, which have necessitated the increased reliance upon NAMs within chemical risk assessments. As in vitro studies become increasingly more common, it is essential to consider in parallel how these data relate to those produced from in vivo systems. Furthermore, it can be beneficial to use data derived through in vitro testing to inform the efficient design of in vivo studies, particular in terms of selecting dose, exposure duration, and target tissues to evaluate. Here we discuss aspects that should be considered and understood when designing NAMs-based studies evaluating air pollution toxicology, specifically focusing on experimental and computational-based methods to better translate in vitro findings into in vivo toxicological understanding.

### 3.1. Experimental Methods to Compare In Vitro Findings to In Vivo Toxicology

It is of utmost importance to design and implement experimental methods that allow for the production of in vitro-derived data that are amenable to informing *in vivo*-level toxicology for air pollution assessments. To achieve such translational data, it is critical to provide consistency across models of test subjects (e.g., cells, animals, humans) when generating test articles for inhalation exposure as the primary route of administration. Some of these similarities include air exchange rate, temperature, relative humidity, exposure generation, and analytical chemistry methods. Some differences between in vitro vs. in vivo models and exposure systems will inevitably persist, and in these instances, it is imperative to understand and account for these differences. For example, the route of exposure administration often varies between study designs. Animals exposed via intratracheal instillation or pharyngeal aspiration (intratracheal) do not always experience the same effective route as animals exposed via inhalation [149]. Additionally, previous studies have utilized direct liquid instillation at the ALI for respiratory cell cultures as a surrogate for inhalation study designs [150]. In vitro designs that more closely parallel in vivo inhalation studies could include gaseous exposure conditions at the ALI, as we have done previously [5,7,51,53,95]. 

**Figure 3:**
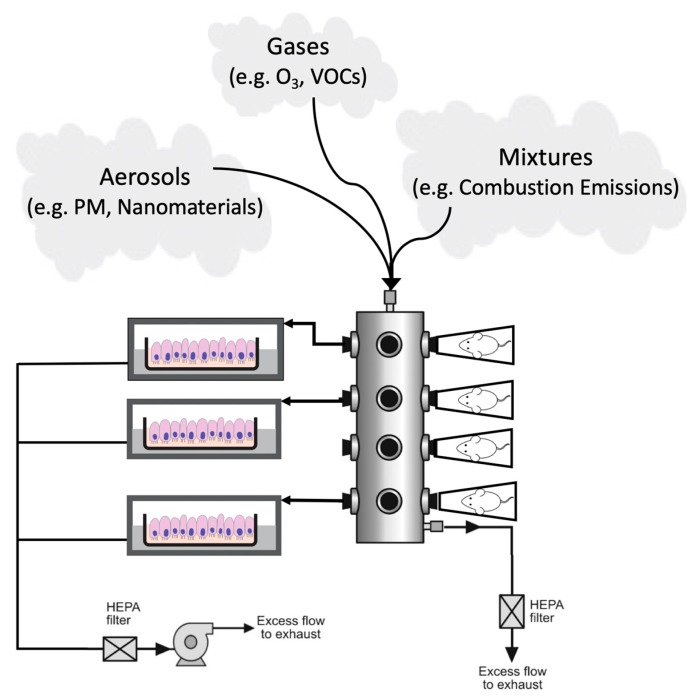
Overview of exposure chamber systems that can be used to more efficiently evaluate in vitro vs. in vivo toxicity responses to air pollutants through laboratory experimentation. These proposed designs allow for the evaluation of rodents exposed to air pollutants using the same exposure generation systems and experimental set-up as parallel in vitro experimentation. Such methods could be used to increase confidence in in vitro data, with the ultimate goal of reducing reliance upon animal testing. Abbreviations: PM, particulate matter; O_3_, ozone; VOCs, volatile organic compounds.

A method to ensure consistency between routes of administration that we propose includes the use of an exposure chamber that is independent of the test model. As illustrated in Figure 3, such an exposure system would allow for either in vitro or in vivo model evaluation using the exact same generation and delivery system. Such design minimizes variables for generation systems, analytical monitoring, and delivery systems that allow for more direct comparisons between in vitro- and in vivo-induced toxicological responses. For in vivo evaluations (comprising animal or human subjects), this exposure system can include either whole-body or nose-only chambers. Whole-body chambers are typically used for gases and vapors while aerosols use nose-only chambers to minimize secondary routes of exposure through the gastrointestinal tract by animal grooming (OECD GD 39). The parallel in vitro evaluations should operate under the same considerations used for in vivo systems but with parameters controlled for the cells in an ALI environment. For example, the operating parameters such as temperature and relative humidity must be controlled to physiological conditions that also maintain cell viability (e.g., 37 °C and 75%–85% relative humidity). In addition, a reduced air flow rate is required to eliminate cell desiccation, but with the caveat that analytical limitations will be introduced [95,99].

As an example, if we consider chamber airflow rate as an important parameter of inhalation exposures, the comparison between a whole-body (*in vivo*) design to an in vitro exposure system is dramatic. A typical 1 m^3^ inhalation chamber operates at 15 air changes per hour or 250 L/min. While a typical in vitro direct perpendicular flow exposure system operates at ~10 mL/min per well (ALI) or for a 24-well cell culture plate ~240 mL/min. This 1000-fold decrease in airflow rate limits many other operating parameters like maintaining relative humidity and quantitative analytical analysis of the test article. Therefore, special humidification systems must be employed to maintain the cell environment (75%–85% relative humidity) without causing condensation in the exposure system. The need to provide quantitative analytical results of the actual exposure concentration at ALI is much more difficult, especially for gases and vapors as typical monitoring instruments operating flow rates used for inhalation chambers will not suffice at airflow rates of 240 mL/min for in vitro exposure systems. There are many solutions to address these in vitro exposure issues but as with in vivo inhalation exposure systems, characterization and qualification of the exposure system must be conducted [151].

### 3.2. Computational In Vitro-To-In Vivo Extrapolation Modeling

The extrapolation of experimental evidence for suitability in human health assessments represents an important aspect of chemical risk evaluation that is of growing interest due to ethical and resource considerations [152,153,154,155]. As discussed previously, traditional toxicology testing methods often involve extrapolation across species and routes. Similar challenges are posed in extrapolating from in vitro testing data to in vivo conditions. Computational-based in vitro-in vivo extrapolation (IVIVE) is necessary despite the potential for using human—rather than animal—biological material in vitro [156,157]. In fact, the National Academies of Sciences, Engineering, and Medicine, as well as many others, have described a “parallelogram approach” in which IVIVE and animal-to-human extrapolation are considered as comparable approaches [158]. Within the parallelogram approach, the four categories of data that make up the “corners” include: (i) “human, in vivo”, (ii) “human, in vitro”, (iii) “animal, in vivo”, and (iv) “animal, in vitro.” Extrapolation is needed to translate data from one “corner” to any of the others. Interestingly, one of the best ways to learn about IVIVE for humans has been to perform it for animals, since in some cases it is easier to obtain both in vitro and in vivo measures of animal toxicity as opposed to human toxicity [159,160,161,162]. 

IVIVE can be sub-divided into the extrapolation of in vitro data towards mechanism of action applications, based upon the interpretation of bioactivity (i.e., toxico- or pharmaco-dynamics); and the extrapolation of in vitro data towards toxico- or pharmacokinetics applications, which inform processes of absorption, distribution, metabolism, and excretion (ADME) of a chemical by the body [156,163]. For volatile compounds, both dynamics and kinetics in vitro can be confounded if not properly accounted for. For example, high-throughput testing designs often make use of multi-well plates for the testing of multiple chemicals per plate that are typically not sealed, allowing volatile chemicals to not only escape a test well (therefore reducing effective concentration) but also to contaminate nearby wells [164,165,166]. However, given appropriate handling, in vitro data can be very useful for informing in vivo predictions: Quick and Shuler (1999) demonstrated a predictive toxicokinetic model for the volatile compound naphthalene that could be constructed using in vitro data on rate constants for metabolic kinetics [167]. This approach was generalized by Jongeneelen and Ten Berge (2010) to construct a chemical-agnostic (“generic”) physiologically-based toxicokinetic (PBTK) model for a range of volatile substances for which metabolic rate constants could be characterized in vitro [168]. Models for kinetic aspects such as dermal evaporation were included by these researchers based purely on physicochemical properties.

To date, in vitro measures of toxicokinetics for volatile compounds have not been included in chemical risk prioritizations [163,169,170] because both bioactivity and kinetic data cannot be obtained using the same methods as previously implemented for the non-volatile and semi-volatile compounds that make up the majority of high-throughput screening libraries [164,171]. The development of a generic PBTK modeling framework that can concurrently handle higher throughput data on semi- and non-volatile chemicals with lower throughput data on volatile chemicals would enable comparison of chemical risk rankings across these diverse chemistries. Such approaches could more effectively link in vitro data from air pollution toxicity tests to chemical risk evaluations.

### 3.3. Current Limitations and Future Directions

This review highlights that the field of in vitro toxicology within air pollution studies is rapidly growing to meet the current demands of twenty-first century exposure science and toxicology. Alongside this growth, there remain some important limitations and data gaps that should be addressed in future research efforts. These data gaps were discussed throughout the current review and include those relevant to instrumental/experimental limitations and capabilities to extrapolate in vitro-derived findings into in vivo-level outcomes. Experimental methods are clearly being expanded upon to more adequately test air pollutant toxicity through the use of several types of in vitro models, as well as advancements in experimental designs to allow for more effective IVIVE. Chemical exposure conditions that have been used to evaluate air pollution toxicity in vitro have historically been limited when testing complex mixtures of volatiles and PM that are more representative of real-world exposure conditions. An additional limitation when evaluating toxicity endpoints using in vitro screening methods is the amount of sample that can be collected within the employed plate designs (e.g., 6-, 12-, 24-, 96-, and 384-well plates). These designs typically yield smaller amounts of DNA/RNA/protein in comparison to full cell culture flasks or tissue samples collected in vivo. However, advances in biotechnologies are allowing for improved sensitivity with smaller sample requirements to extract biological data with increased efficiency. Computationally, generic PBTK modeling frameworks within the field of inhalation toxicology would prove beneficial towards increasing our understanding surrounding the dosimetry of larger numbers of chemicals present in air pollution. These current limitations and data gaps should be addressed as this field of research continues to expand.

## 4. Conclusions

In conclusion, there is clear interest towards increasing reliance upon in vitro screening within air pollution toxicity and risk assessment evaluations. This review provides a timely discussion on the expanding in vitro approaches that can be implemented to evaluate toxicity resulting from various air pollutant exposure conditions. Critical aspects of experimental design and data interpretation that are highlighted include cell models, cell exposure conditions, exposure chambers, and toxicity endpoints. The integration of in vitro findings into informing in vivo toxicology and overall human health risk assessment is also discussed in the context of IVIVE and other extrapolation approaches. Though this field has expanded in recent years, there remain data gaps and future research directions that can be addressed with the goal of maximizing testing efficiency and data utility from such experimental efforts. Taken together, this review summarizes important aspects to consider when designing, conducting, and interpreting NAMs within the field of air pollution research.

## Figures and Tables

**Table 1:** Overview of cell models available for air pollution toxicology studies. Example model categories are listed alongside advantages and disadvantages towards the evaluation of toxicity associated with air pollutant exposures.

Cell Model Category	Advantages	Disadvantages
**Monoculture cell lines**	- Easy to grow and maintain - Inexpensive - Amenable to high-throughput screening - Reproducible toxicity responses - High viability in comparison to other models - Available for different cell types present in the respiratory tract - Availability/standardization allows for comparison of results among different groups	- From one donor, which does not account for population response variability - In cancer/transformed cell lines, genetic and epigenetic profiles differ from non-cancer cells - Depending on cell line, limited representation of an in vivo epithelial barrier - Findings are limited to one cell type
**Cells from human donors**	- Allows for evaluation of specific subpopulation of interest (e.g., age, disease, sex, etc.) - Allows for identification of cell populations with increased susceptibility to adverse effects - Improved physiological relevance - Evaluation of responses across multiple cell types - Can be maintained in culture for weeks/months at a time - Can be used for repeated exposures to simulate chronic conditions	- Expensive - Requires more advanced cell culture capabilities - Time and resource intensive to process and maintain cell culture - Difficult to determine which cell type drives observed toxicity
**Lung-on-a-chip**	- Improved physiological relevance due to potential cell-to-cell communications - Continuous replenishment of nutrients and removal of waste - Can model influence of circulating immune cells - Includes physical and mechanical properties involved in in vivo pulmonary functions - Can allow for organ-crosstalk (e.g., body-on-a-chip)	- Difficulties surrounding ease of use - Expensive - More chronic exposures are currently difficult due to viability considerations - Technologies are more recently developed and may require further testing - Insufficient biological material for downstream analyses
